# Time and contamination level dependence of metal bioaccumulation and multibiomarker responses in fish: implications for biomonitoring

**DOI:** 10.1007/s10661-026-15181-9

**Published:** 2026-03-23

**Authors:** Paloma Kachel Gusso-Choueri, Rodrigo Brasil Choueri, Luciane Alves Maranho, Aline Vecchio Alves, Caio Rodrigues Nobre, Gabriela Pustiglione Marinsek, Renata de Britto Mari, Tailisi Hoppe Trevizani, Rubens Cesar Lopes Figueira, Denis Moledo de Souza Abessa

**Affiliations:** 1 Laboratório de Ecotoxicologia-Unisanta, Universidade Santa Cecília, R. Oswaldo Cruz, 277–CP 11045-907-Boqueirão, Santos-SP, Brazil; 2 Campus Do Litoral Paulista, Universidade Estadual Paulista Júlio de Mesquita Filho-Unesp, Praça Infante Dom Henrique, S/N, CP 11330-900 São Vicente, SP Brazil; 3 Departamento de Ciências Do Mar, Universidade Federal de São Paulo, Campus Baixada Santista. Rua Maria Máximo, 168-Ponta da Praia-Santos/SP-CEP: 11030-100, Santos, Brazil; 4 Laboratório Morfofuncional, Universidade de Ribeirão Preto (UNAERP), Avenida Dom Pedro I, 3.300, 11440-003 Guarujá, São Paulo, Brasil; 5 Laboratório de Química Inorgânica Marinha. Pça Do Oceanográfico, Universidade de São Paulo, Instituto Oceanográfico, 191, Cidade Universitária, 05508-120 São Paulo, SP Brazil

**Keywords:** Sediment, Biomarkers, Histopathology, Bioaccumulation, Ecotoxicology

## Abstract

This study evaluated how different levels of sediment metal contamination (ranging from below to above toxicity thresholds) and exposure durations (3–14 days) influence metal bioaccumulation and biomarker responses in fish under estuarine conditions. Using an integrated biomarker approach, the relationships among environmental contamination, metal accumulation, and fish health were examined to improve biomonitoring strategies. At the highest contamination level, 100% mortality was observed. Under low contamination, fish eliminated excess Cu, Zn, and Hg, whereas moderate contamination resulted in progressive accumulation of Cu and Zn; Pb accumulated under both exposure levels. Early biomarker responses included decreased GSH, inhibited GPx, increased lipid peroxidation, histopathological liver lesions, and neurotoxicity, followed by partial recovery. DNA damage increased over time and across contamination levels, likely due to impaired antioxidant defenses and/or direct metal–DNA interactions. Histopathological alterations were more strongly associated with sediment contamination than with tissue concentrations, indicating that body burdens may not fully represent exposure. GSH depletion and DNA damage were the most consistent biomarkers, whereas LPO and histopathology were sensitive primarily at early exposure stages. These findings reinforce the importance of considering both exposure duration and contamination level when interpreting biomarker responses and highlight the limitations of short-term or single-biomarker assessments. Biological effects detected at low to moderate contamination support the need for more precautionary sediment-quality thresholds. Overall, integrating biochemical, physiological, and histological biomarkers provides early detection of sublethal effects and a comprehensive assessment of ecosystem health. Consequently, a multibiomarker framework represents a powerful tool to strengthen the monitoring, evaluation, and management of aquatic environmental quality.

**Supplementary Information:**

The online version contains supplementary material available at 10.1007/s10661-026-15181-9.

## Introduction

In quality assessments and monitoring programs of aquatic environments (marine, freshwater, or transitional environments such as estuaries), it is generally assumed that exposure levels determine the accumulation of metals in the soft tissues of aquatic organisms, and consequently, the biological effects these metals may cause (van der Oost et al., 2003; Luoma & Rainbow, 2005; Wang & Rainbow, 2008; Zahran, et al., 2025). However, for fish exposed to moderate contamination, several studies have reported no clear relationship between contaminant concentrations in sediments and soft tissues and the resulting biological responses (Abdolahpur et al., 2013; Monroy et al., 2014; Gusso-Choueri et al., 2022). This lack of association is likely related to the influence of multiple toxicokinetic and toxicodynamic mechanisms, which shape the cause-and-effect links between environmental exposure and bioaccumulation (conceptually defined as the increase in contaminant concentrations in aquatic organisms through uptake from the surrounding medium; Wang, 2016), as well as between bioaccumulation and biological responses. Advancing the understanding of these complex interactions is essential for improving the interpretation of ecotoxicological data in quality assessment and for strengthening environmental monitoring across aquatic ecosystems, from estuarine and marine to freshwater environments (Santhosh et al., 2024; Zahran, et al., 2025).

Aquatic organisms have a wide diversity of strategies to deal with metals after their absorption, which vary within a spectrum from maximum excretion to maximum accumulation. Therefore, for the same environmental exposure, there may be large differences in metal concentrations in the soft tissues of different species (Bhat et al., 2025; Rainbow, 1998, 2002). Furthermore, the bioaccumulation of a specific metal does not depend only on the level of exposure but also is related to environmental conditions that interfere with the bioavailability as well as the biochemical and physiological processes using which a specific organism deals with the internal levels of that metal (Allen-Gil & Martínov 1995; Luoma & Rainbow, 2008; Terra et al., 2008; Copat et al., 2012; Salam et al., 2020; Wang et al., 2025). Metal accumulation also depends on the organism’s ecology (e.g., feeding habits and trophic level), the intensity of contact between the organism and the pollutant (e.g., filtration rate in invertebrates, ingestion rate, and gill ventilation), how much of the pollutant is effectively taken up (e.g., quality of digestive enzymes and tissue permeability) and, finally, detoxification strategies (storage, detoxification, or elimination) (Wang & Rainbow, 2008). Still, metal uptake and elimination rates may not necessarily be constant over the exposure time (Adams et al., 2011).

Therefore, without knowing in detail the mechanistic relationship between environmental exposure, internal levels, and toxic responses to metals, it is difficult to predict the health of aquatic organisms based on environmental or soft tissue concentrations of contaminants. Nevertheless, some studies have sought to associate levels of contaminants in tissues and toxicity (Oliva et al., 2014; Barhoumi et al., 2014; Morachis-Valdez et al., 2015; Souza et al., 2018; Gusso-Choueri et al., 2015, 2016, 2022). It is logical to expect that the greater the exposure to contamination (concentration rate and/or duration time), the greater the manifestation of its effects (as expressed by the classic Haber’s rule) (Gaylor, 2000). Furthermore, a simplified linear etiology is commonly established in which responses at lower levels of biological organization (e.g. molecular, subcellular, and cellular) are considered early, sensitive responses, which precede those at higher levels of biological organization (tissue, systemic, individual, and so on) (e.g., Bayne et al., 1985). However, especially under field conditions, observations do not always fully meet this expectation (e.g., Beyer et al., 1996; Diop et al., 2022; Gusso-Choueri et al., 2015).

Although increasing complexity has been incorporated into the establishment of environmental quality standards or benchmarks for metals—such as the use of toxicity-modifying factors (e.g., corrections based on the Biotic Ligand Model (BLM)) in the USA, Europe, Canada, and Australia/New Zealand (Van Genderen et al., 2020)—toxic responses are still largely based on the premise that the exposure concentration (in water, sediment, or diet) is proportional to the whole-body concentration at the receptor, where toxic action occurs (Meador, 2015). To some extent, this premise is useful for representing ecotoxicological risks associated with chemical contaminants, particularly under conditions of low or high contamination. This premise, however, as a consequence of the complexity of pollutant–receptor interactions, carries a conceptual imprecision that may confound environmental diagnoses, mainly at moderate contaminant concentrations. A clearer understanding of the relationships among environmental metal concentrations, tissue burdens, and toxic effects is therefore essential for improving environmental management and assessment tools (Meador et al., 2008a, 2008b; Dyer et al., 2011; Zharan et al., 2025), especially in estuarine systems, where natural stressors may mask contaminant-related effects and complicate biomonitoring based on biomarker responses (Nagarajan et al., 2022; Truchet et al., 2021).

Biomarkers can provide mechanistic information linking soft tissue concentrations of pollutants to their associated toxic effects (Hwang et al., 2008; Almeida et al., 2024; Bhat et al., 2025). Biomarker responses at different levels of biological organization (subcellular, cellular, and tissue), when integrated with chemical analyses, can reduce much of the variability observed in toxic thresholds for a given contaminant (Meador et al., 2008a, 2008b), thereby decreasing uncertainty in cause-and-effect relationships between exposure and biological responses.

The mechanisms of trace metal toxicity involve the generation of reactive oxygen species, oxidative stress, and genotoxicity (Kumar et al., 2024; Livingstone, 2003). Accordingly, biochemical biomarkers that reflect antioxidant defenses (e.g., reduced glutathione levels and the activities of glutathione S-transferase and glutathione peroxidase), oxidative damage (lipid peroxidation), and genotoxic effects (e.g., DNA strand fragmentation) are sensitive indicators of both exposure to and effects of trace metals (van der Oost, 2003; Gusso-Choueri et al., 2015, 2016). Acetylcholinesterase (AChE) activity is also an important biomarker of trace metal toxicity, as metals can interact with acetylcholine receptors and thereby affect AChE activity (De Lima et al., 2013; Bainy et al., 2006; Romani et al., 2003; Almeida et al., 2024). In addition, AChE activity has been associated with caspase activity (Zhang et al., 2002), which plays a central role in the cascade of programmed cell death events (Li & Yuan, 1999; Wolf & Green, 1999). At a higher level of biological organization, tissue-level responses (i.e., histopathology) represent a link between biochemical and individual-level responses (Gusso-Choueri et al., 2022), providing a more integrative assessment of effects while still allowing the anticipation of apical responses (e.g., growth, reproduction, behavior, and survival).

*Poecilia reticulata* (Cyprinodontiformes: Poeciliidae) is recognized by the Organization for Economic Cooperation and Development (OECD, 1992) as a key species for ecotoxicological studies. This species also has excellent potential for use as a biomonitor in the field of environmental management, environmental quality, and risk assessments, due to its wide geographic distribution, colonization of different habitats (from freshwater to estuarine), and its presence in highly degraded environments (Rocha et al., 2015). As a tolerant species, it is important that studies assessing contamination responses in *P. reticulata* be based on early-warning, sensitive endpoints selected in light of toxicodynamic mechanisms, thereby allowing management decisions to be made in a conservative and preventive manner. The application of biomarker-based indicators, spanning molecular to histological levels, should be further promoted in environmental monitoring, as their high sensitivity supports a preventive rather than a corrective approach to pollution management (Pereira et al., 2014). In Europe, the Marine Strategy Framework Directive (MSFD) has incorporated biomarkers into its guidelines, but emphasizes the need for additional research to clarify their practical contribution to environmental quality assessment (Zampoukas et al., 2014). Empirical studies addressing the effectiveness of biomarkers within the MSFD framework remain limited (Capela et al., 2016; Dallas & Jha, 2015; Giltrap et al., 2017; Gusso-Choueri et al., 2022).

 The present study aimed to assess contamination levels and the time-dependent patterns of bioaccumulation and biomarker responses in *P. reticulata* to metals in sediments, using a multilevel, integrated biomarker approach to provide insights into the suitability of this species as a biomonitor for estuarine environments. It is hypothesized that tissue accumulation and ecotoxicological responses do not follow a linear relationship with exposure (i.e., sediment metal concentrations or exposure time), suggesting that, although Haber’s rule provides a general prediction of the exposure–accumulation–response relationship, it may not hold when considering biomarker responses in *P. reticulata* exposed to moderate metal concentrations in sediments. Nevertheless, the severity of injuries is expected to increase with both the level of contamination and the duration of exposure. The assessment of correspondences, divergences, and complementarities between exposure levels (contamination level and exposure time) and biological responses (metal concentrations in soft tissues, biochemical responses, and histopathological alterations) supports the use of integrated biomarkers in *P. reticulata* as a valuable tool for environmental monitoring and quality assessment in estuarine ecosystems.

## Material and methods

### Experimental design

Female individuals of *P. reticulata* were exposed, under laboratory conditions, to sediments spiked with a mixture of metals (inorganic Cu, Pb, Zn, and Hg) at four contamination levels (negative control, low, moderate, and high contamination) in independent experiments lasting 3, 7, and 14 days. Mortality was assessed throughout the experiments. Bioaccumulation of Cu, Pb, Zn, and Hg (in the whole organism), biochemical biomarkers of antioxidant activity (levels of reduced glutathione and activities of glutathione S-transferase and glutathione peroxidase) and effects (levels of DNA damage and lipid peroxidation) in the liver, a neurotoxicity biomarker in the axial muscle (acetylcholinesterase activity), and histopathological responses in the liver were evaluated in organisms that survived until the end of the experiments.

All treatments, including controls, were conducted using two 15-L aquariums per treatment, each containing 12 individuals, totaling 24 individuals per treatment. At the end of the experiment, individuals were mixed and randomly distributed among the different analyses, such that the observed variability includes both individual-level biological variation and potential aquarium-associated effects (although effects at the aquarium level and between-aquarium variance could not be explicitly estimated under this experimental approach).

### Sediment collection, storage, and initial characterization

The sediments used in all tests were collected in a reference area, the mouth of the Itaguaré River (−23.763° S and −45.773° W) (São Paulo, Brazil), which is a confluence of two legally protected areas. Previous studies have also demonstrated that this area does not present metal contamination (Altafim et al., 2023). The collected sediments were transported, cooled in a plastic container, and stored in the laboratory in the dark, at a constant temperature of 4 °C.

A ratio of 20% muddy sediment and 80% sandy sediment was used, with grain size previously determined by wet sieving (Mudroch & Macknight, 1994) and classified based on the Wentworth scale. The calcium carbonate (CaCO_3_) content was quantified through the addition of HCl (5N) (Hirota & Szyper, 1975) and the organic matter (OM) content was quantified using the ignition mass loss method (Luczak et al., 1997).

### Sediment spiking

Data from estuarine environments show a high frequency of occurrence of Cu, Zn, Hg, and Pb at levels possible or likely to cause damage to biota (CETESB, 2005; Choueri et al., 2009; Abreu et al., 2016; Kim et al., 2016; Moreira et al., 2017). This mixture of metals was tested at the concentrations presented in Table 1. The test concentrations of each metal were established in accordance with the guideline values established in Brazilian legislation for characterizing the quality of dredged material (Conama Resolution no. 454/2012) (Brazil 2012). This resolution proposes two levels of action, with level 1 being the threshold for possible effects and level 2 for probable effects. In this legislation, the guiding values for levels 1 and 2 of Cu, Pb, and Zn were taken from Long et al. (1995) while for Hg, level 1 was established according to Environmental Canada and Ministère du Développement durable, de l'Environnement et des Parcs du Québec (2008) and level 2 was established according to Hamburg Port Authority (2011). Thus, (a) the low contamination level used in this study had final concentrations of metals below level 1 of Conama 454 (75% of values); (b) the moderate level had final concentrations of spiked metals between levels 1 and 2 of Conama 454 (average between values from levels 1 and 2); and (c) the high contamination level had metal concentrations above level 2 of Conama 454 (110% of values). The exposures to metal-spiked sediments were accompanied by exposures to unfortified sediments, whose responses represented the negative control of contamination.

**Table 1:** Nominal concentrations of spiked substances in test sediments (mg kg^−1^). “Low” contamination represents values below level 1 of Conama 454, “moderate” contamination represents metal values between levels 1 and 2 of Conama 454 (average between values of levels 1 and 2) and “high” contamination represents concentrations of metals above level 2 of Conama 454 (110% of values)

Metal	Sediment control	[Low]	[Moderate]	[High]
Copper (CuSO_4_)	Background concentration	25	152	297
Lead (PbCl_2_)		35	132	240
Zinc (ZnSO_4_)		112	280	451
Mercury (HgCl_2_)		0.23	0.65	1.10

Sediment spiking was carried out using a stock solution prepared by dissolving metal standards (Sigma-Aldrich®) in dilution seawater (seawater reconstituted from mixing distilled water and commercial sea salt Red Sea® until reaching a salinity of 14). The contaminated sediment was homogenized for 10 min. After homogenization, the sediment was kept at 4 °C and protected from light for 7 days so that the added metals could reach chemical equilibrium with the sediment (ASTM, 2014). Aliquots of these sediments were separated and stored in plastic packaging for metal quantification.

### Bioassays

The test organisms were acquired from a fish farm and taken to the laboratory where they were acclimatized in 500-L tanks for approximately 15 days. Salinity was adjusted from 0 to 14 gradually to reach an estuarine salinity. Once the final salinity was reached, the organisms were kept for 10 days under these conditions. Physical–chemical parameters (non-ionized ammonia, nitrite, salinity, dissolved oxygen, and pH) were monitored throughout acclimatization, and photoperiod (12 h:12 h) and temperature (25 ± 2 °C) were kept constant. The individuals were daily fed with Alcon Basic® aquarium fish food and no mortality during the acclimatization period was recorded.

The study has been reviewed and approved by the Ethics Committee of *Universidade Estadual Paulista*, Institute of Biosciences, Coastal Campus of São Paulo under Protocol No. 12/Z017-CEUA, and throughout its development, compliance with all ethical and animal welfare requirements has been ensured. The tests were carried out as proposed by Costa et al. (2009) and USEPA (2000). In each test system with a total capacity of 15 L, 2 L of sediments were placed and 10 L of dilution seawater (salinity of 14) were gently added, avoiding resuspension. Individuals (*n* = 12) of homogeneous size (6.0 cm ± 0.5) were introduced into the aquariums (*n* = 2) after a 48-h stabilization period of the test system.

The animals were fed every two days with commercial fish food (Alcon Basic®) throughout the exposure period. All aquariums had constant and gentle aeration (to avoid sediment resuspension), had parameters measured at the beginning and end of the exposure period (temperature of 22 °C ± 0.3, pH of 8.0 ± 0.1, salinity of 14 ± 2, and dissolved oxygen of 5.24 mg L^−1^ ± 0.5) and photoperiod set to 12 h:12 h light:dark. The individuals were assessed for mortality throughout the experiments, and the total number of dead or immobile organisms was recorded at the end of the exposure period. 

Prior to exposure, 21 organisms from the same batch of individuals were also randomly separated, sedated, and euthanized to analyze the conditions of the organisms before the test (T0). At the end of the tests, the organisms were sedated with benzocaine (0.1%), weighed, measured, and euthanized through spinal section. Each group of 24 organisms (12 individuals × 2 aquariums) exposed under each treatment was randomized. Due to the small size of the organisms and the consequent limitation in obtaining sufficient tissue mass for the proposed analyses, pools of three and four individuals were established for tissue concentration and biochemical biomarker analyses, respectively. Individuals used for liver histopathological analyses were not pooled. Individuals (whole organism) were separated for the analysis of metal body burdens (*n* = 1 pool of three individuals), and the remaining organisms were dissected to remove the liver and axial muscles for biochemical biomarker (*n* = 4 pools of four individuals each) and histopathological (*n* = 5 individuals) analyses. The same biomarker analyses were performed on these individuals to allow comparison between pre-exposure and post-exposure conditions.

### Biological response analyses

#### Biochemical biomarkers

Liver and axial muscle tissues were kept on ice and homogenized in pools of four individuals at 20% mass:volume (m:v), in Tris-HCl buffer (50 mM Tris; 1 mM EDTA; 1 mM DTT; 50 M sucrose; KCl at 150 mM, 1 mM PMSF, pH 7.6). Aliquots of liver homogenates were separated for quantification of DNA damage and lipid peroxidation (LPO). The remainder of the sample was centrifuged at 12,000 g for 20 min at 4 °C, and aliquots of this supernatant were separated for analysis of glutathione-S-transferase (GST) activity, glutathione peroxidase (GPx) and quantification of non-protein thiols (reduced glutathione, GSH). In the case of muscle tissue, the supernatant fraction was used to determine acetylcholinesterase (AChE).

The activities of GST (Keen et al., 1976) and GPx (Sies et al., 1979) were determined by spectrophotometry at 340 nm. GSH levels were measured spectrophotometrically at 415 nm (Sedlak & Lindsay, 1968). Analysis of AChE activity was quantified at 415 nm using the colorimetric method proposed by Ellman et al. (1961). Lipid peroxidation (LPO) levels were quantified by means of the thiobarbituric acid reactive substances (TBAR) method (Wills, 1987) using fluorescence (λex 532 nm and λem 556 nm). The method used to assess DNA damage was alkaline precipitation (Gagné and Blaise, 1995). The assay is based on the precipitation of genomic DNA bound to proteins using sodium dodecyl sulfate (SDS), leaving single breaks in the DNA–protein chain free in the supernatant. These DNA strands were quantified using fluorescence (λex 360 nm and λem 450 nm) after staining with Hoescht® dye. Salmon sperm DNA standard solutions were used for calibration.

All analyses were normalized by protein concentrations, which were determined by spectrophotometry at 595 nm (Bradford, 1976) using bovine serum albumin protein (BSA) as standard. Absorbance and fluorescence readings were performed on a microplate reader (Biotek-Synergy ™ HT).

#### Liver histopathology

Liver samples were dissected, separated into histological cassettes, and fixed in ALFAC (alcohol, formaldehyde, and glacial acetic acid) for 16 h. The material was kept at 4 °C in 70% alcohol until the preparation of permanent slides.

To prepare the slides, the fixed liver samples were dehydrated, then cleared and included in Paraplast Plus resin (Sigma®). Then, 5-µm sections were then obtained and, after a new clearing and subsequent hydration, stained in hematoxylin/eosin. The assessment of liver damage was carried out following the method proposed by Bernet et al. (1999), with modifications. For each change, an importance factor (*w*) was assigned according to the degree of reversibility, being (1) for changes that are easily reversible, (2) for moderate changes, potentially reversible with the end of exposure, and (3) for irreversible changes, which lead to partial or total loss of organ function. To obtain the index of histopathological changes (histopathological index or HI), the importance factor was multiplied by a second numerical value (a) according to the extent or frequency of the lesion (ranging from 0 to 6, with 0 = absent and 6 = abundant/extensive).

#### Chemical analysis

Samples of whole sediment and whole organisms were freeze-dried (K105 freeze dryer, Fisyka Biotechnology, Liobras, Brazil). The samples of sediments separated for determination of Cu, Pb, and Zn were subjected to acid digestion using the SW846 USEPA 3050B method (USEPA, 1996), where 5 mL of HNO_3_, 2.5 mL of H_2_O_2_ are added to an aliquot of 1 g of dry sediment (30% V/V), and 5 mL of HCl, and the mixture is heated to 90 °C. The samples were filtered and diluted in 50 mL of ultrapure water (Milli-Q). For extraction of metals from the organisms, the methodology used was adapted from Trevizani et al. (2016). Cu, Pb, and Zn were analyzed by inductively coupled plasma optical emission spectroscopy (ICP-OES) (Varian equipment, model 710ES) following the USEPA 6010c (2007) protocol. Mercury analysis was performed following the USEPA 7471B method (USEPA, 1994). After the extraction process, the concentrations of mercury extracted from the samples were analyzed in an ICP-OES with a vapor generation accessory (VGA) attached.

For all quantifications, quality controls were carried out based on sample fortification and blank analysis. Acceptable recovery rates ranged from 75 to 125% (USEPA, 1996).

### Data treatment

Data analysis was organized into three distinct steps: (i) evaluation of potential laboratory effects on the general health condition of the tested organisms by comparing the status of biomarkers before (T0) and after the bioassays (organisms from the control treatment, after 3, 7, and 14 days of testing); (ii) assessment of the effects of metal contamination in the sediment on each biomarker response individually after 3, 7, or 14 days of exposure; and (iii) integration of all variables by assessing associations between biomarker responses (GSH, GST, GPx, AChE, LPO, DNA damage, and histopathological index), metal body burdens, and sediment concentrations (for Cu, Pb, Zn, and Hg).

The assessment of laboratory effects on the general health condition of the organisms during the test was done by multivariate PERMANOVA analysis. One fixed factor with 2 levels was established for all the biomarkers: (i) before (data from T0) and (ii) after 3, 7, or 14 days of the test (data from each respective sediment control treatment). After normalization of the variables, the resemblance matrices were generated using Euclidean distance. Post hoc tests were performed when significant effects were detected by the main test. Monte Carlo *p*-values were used in instances where the number of permutations was lower than 50, and the residuals were permuted using unrestricted permutation of raw data (Anderson et al., 2008).

The effect of metal contamination on each biomarker within each exposure time was evaluated by means of univariate analyses of variance. All tests were preceded by a check of normality (Shapiro–Wilk’s test) and homoscedasticity of the data (Levene’s test). In cases where the assumptions of parametric tests were met, one-way ANOVA tests were performed, complemented by Dunnet’s post hoc test; otherwise, Kruskal–Wallis (KW) tests and Tukey’s test were used as post hoc. The level of the contamination factor corresponding to “high contamination” was not included in the analyses since there were no living organisms left after the exposure time. The significance level was set at 0.05 for all analyses of variance. 

Finally, all variables (biomarker responses, metal levels in organisms and sediments) were assessed together using factor analysis with extraction by principal component analysis (FA/PCA) to highlight the associations between biomarker responses (GSH, GST, GPx, AChE, LPO, DNA damage and histopathological index), metal body burdens, and levels of metals in sediments (Cu, Pb, Zn, and Hg). A score of  ≥ 0.50 was used, more conservative than the value suggested by Tabachnic and Fidell (1996). The relevance of the associations observed for each treatment (sediment control, low and moderate concentrations over the three times) was estimated by calculating the factor score of the centroid of all these treatments in relation to the original data. The analyses were carried out using the Statistica® 12 software.

## Results

### Sediment characterization and metal contents

The sediments presented a proportion of 23.12% mud (± 6.17) and 72.64% sand (± 6.27). The organic matter content was 2.27% (± 0.14) and CaCO_3_, 1.97% (± 0.24). As can be seen in Table 2, the metal concentrations analytically quantified in the sediments are very close to the spiked concentrations (Table 2), thus validating the pre-established metal contamination gradient.

**Table 2:** Measured concentrations of the metals spiked in the test sediments (mg kg−1) and organisms at the different levels of contamination and exposure time. The high contamination treatment was not carried out for exposure times 3 and 7 days

Matrix	Metal	3 days			7 days			14 days			
		Sediment control	[Low]	[Moderate]	Sediment control	[Low]	[Moderate]	Sediment control	[Low]	[Moderate]	[High]
Whole sediment	Cu	2.85	10.11	106.82	0.65	28.85	125.42	1.14	27.00	111.13	196.62
	Pb	5.34	15.15	79.54	1.64	24.92	101.09	2.91	36.58	94.36	260.84
	Zn	20.86	40.58	192.31	7.92	88.33	217.04	6.28	95.78	207.46	434.70
	Hg	0.090	0.292	1.215	0.008	0.606	1.100	0.065	0.440	1.182	NM

*NM* not measured

### Bioaccumulation of metals in organisms

**Fig. 1:**
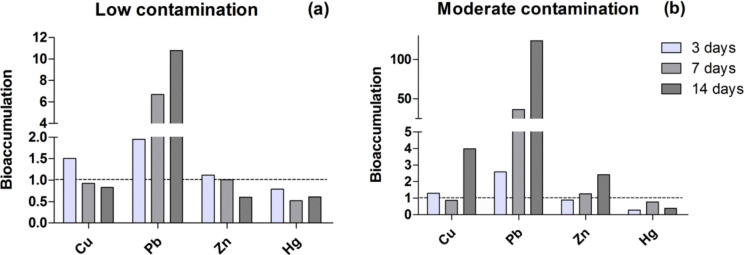
Metal bioaccumulation in the whole organism, expressed as body burden values in exposed organisms normalized to the corresponding body burdens measured in organisms from the respective sediment control. The dashed line at 1.0 represents the reference condition (sediment control), at the two contamination levels tested, low (A) and moderate (B). Values above or below 1.0 indicate proportional increases or decreases in bioaccumulation relative to the control

The results of metal bioaccumulation in the whole organism (i.e. body burdens in exposed organisms in relation to body burdens in organisms in each respective sediment control) (Fig. 1) shows that, in the low-contamination scenario, Cu and Zn were bioaccumulated after 3 days but depurated after 7 and 14 days, ending the exposure time, in the latter case, with concentrations of these metals even lower than those of the sediment control organisms. Increased bioaccumulation over time occurred for Cu, Pb, and Zn at moderate concentrations and, for Pb, at low concentrations as well. Mercury presented a pattern distinct from other metals. In organisms exposed to both low and moderate contamination, Hg levels were lower than those exposed to sediment control.

### Multilevel biomarker responses

#### General health condition of organisms in sediment control

The results of the analyses of biochemical biomarkers and histopathological indices for organisms from T0 (before the exposure time) and exposed to the control sediment for 3, 7, or 14 days (the longest duration among the bioassays) are presented in table S1 (supplementary material). The multivariate structure of biomarker data from organisms exposed to the control sediments at any exposure time did not differ from that of T0 organisms (PERMANOVA; 3 days: Pseudo-*F*(1,9) = 0.23; *p*(perm) = 0.66; 7 days: Pseudo-*F*(1,8) = 0.54; *p*(perm) = 0.51; 14 days: Pseudo-*F*(1,11) = 1.14; *p*(perm) = 0.29), indicating that there was no significant effect of the experimental conditions on the general health condition of the control organisms during the exposure time.

#### Acute effects

Sediments spiked with the highest levels of metals (i.e., above level 2 of Conama 454/2012) caused an acute effect (100% mortality) after 6 h of exposure. Low and moderate contamination levels did not show significant mortality in relation to the control treatment (> 20%) in any of the bioassays (3, 7, and 14 days).

#### Antioxidant activity

**Fig. 2:**
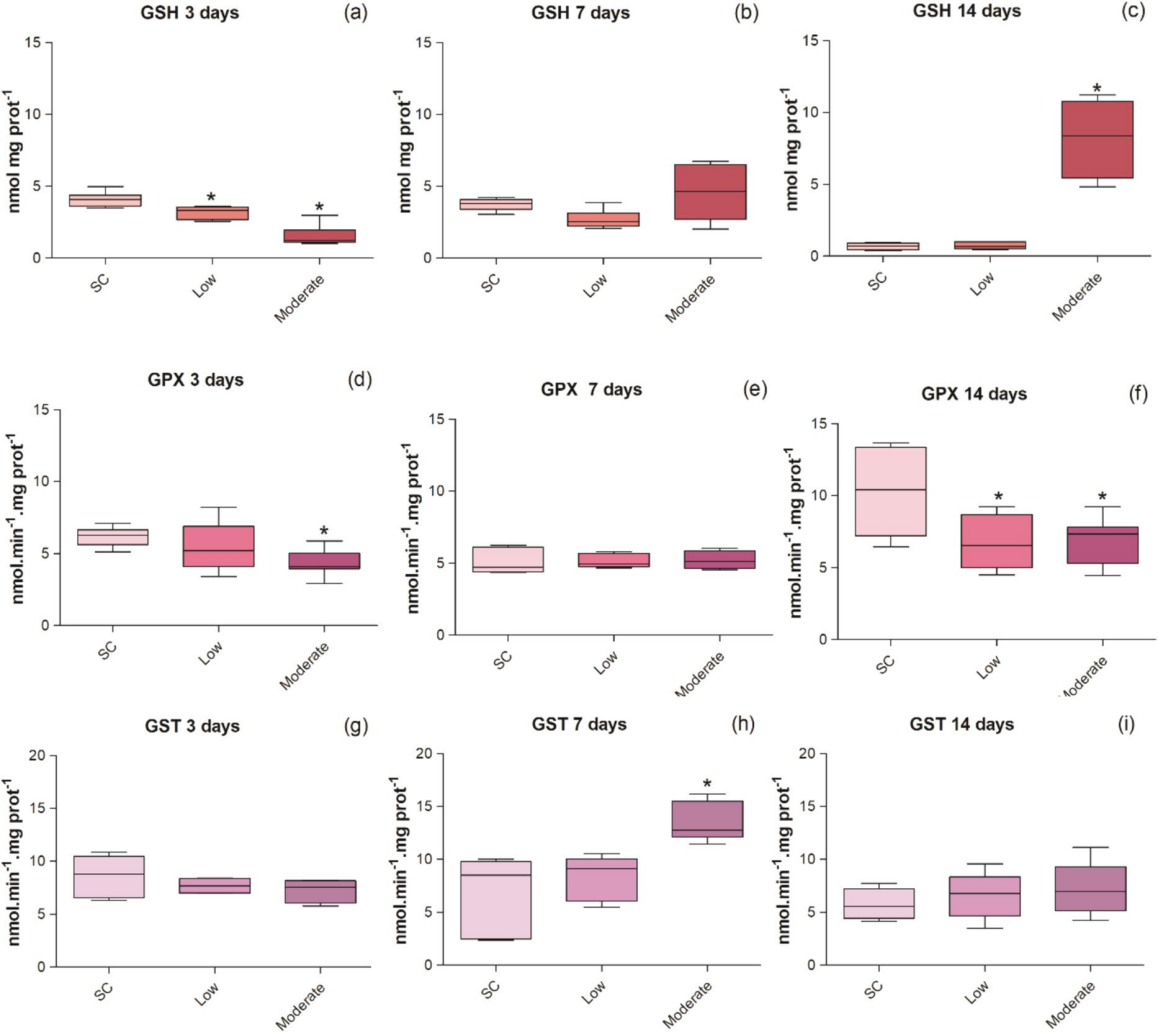
GSH levels (a, b and c) and activities of GPx (d, e and f) and GST (g, h and i) in the liver of *P. reticulate* exposed to different levels of sediment metal contamination and exposure times. Data are presented as boxplots with limits indicating 25 and 75 percentiles; a line inside the box marks the median value; markings below and above the box indicate the 10th and 90th percentiles. The asterisks mean statistical differences compared to the control sediment (*p* < 0.05). SC = sediment control; Low = low metal contamination; Moderate = moderate metal contamination

The levels of GSH and activities of GPx and GST across the levels of metal contamination in the sediment at different exposure times are shown in Fig. 2.

The levels of GSH in the liver of individuals exposed (Fig. 1) to contaminated sediments tended to decrease at both concentrations tested (low and moderate) at the shorter term exposure (3-day test) (ANOVA; *F*(2,19) = 40.75; *p* < 0.0001). At 7-d exposure and after, results for individuals exposed to the low concentration tend to be reduced and to be closer to the control treatment (KW; *H*(2) = 4.90; *p* = 0.09), as well as an increase in the levels of this nonprotein thiol in moderate contamination being recorded as the exposure time got longer (ANOVA; *F*(2,13) = 46.35; *p* < 0.0001).

When comparing the results of GPx activity in the liver of *P. reticulata* exposed to contaminated sediments with the control treatment, an inhibition in the activity of this enzyme was observed at moderate concentrations in bioassays lasting 3 days (ANOVA; *F*(2,20) = 5.06; *p* = 0.02) and 14 days (ANOVA; *F*(2,20) = 4.87; *p* = 0.02). At 14 days of exposure, even individuals exposed to low metal concentrations showed an inhibition in GPx activity (Fig. 1).

GST activity in *P. reticulata* liver tissues only differed significantly from the control treatment at the moderate concentrations of the 7-d bioassays (ANOVA; *F*(2,13) = 8.90; *p* = 0.004). One can also notice, although without significant difference, a slight inhibition at 3 days, followed by an induction at 14 days in this same treatment (Fig. 2).

#### Effect biomarkers

**Fig. 3:**
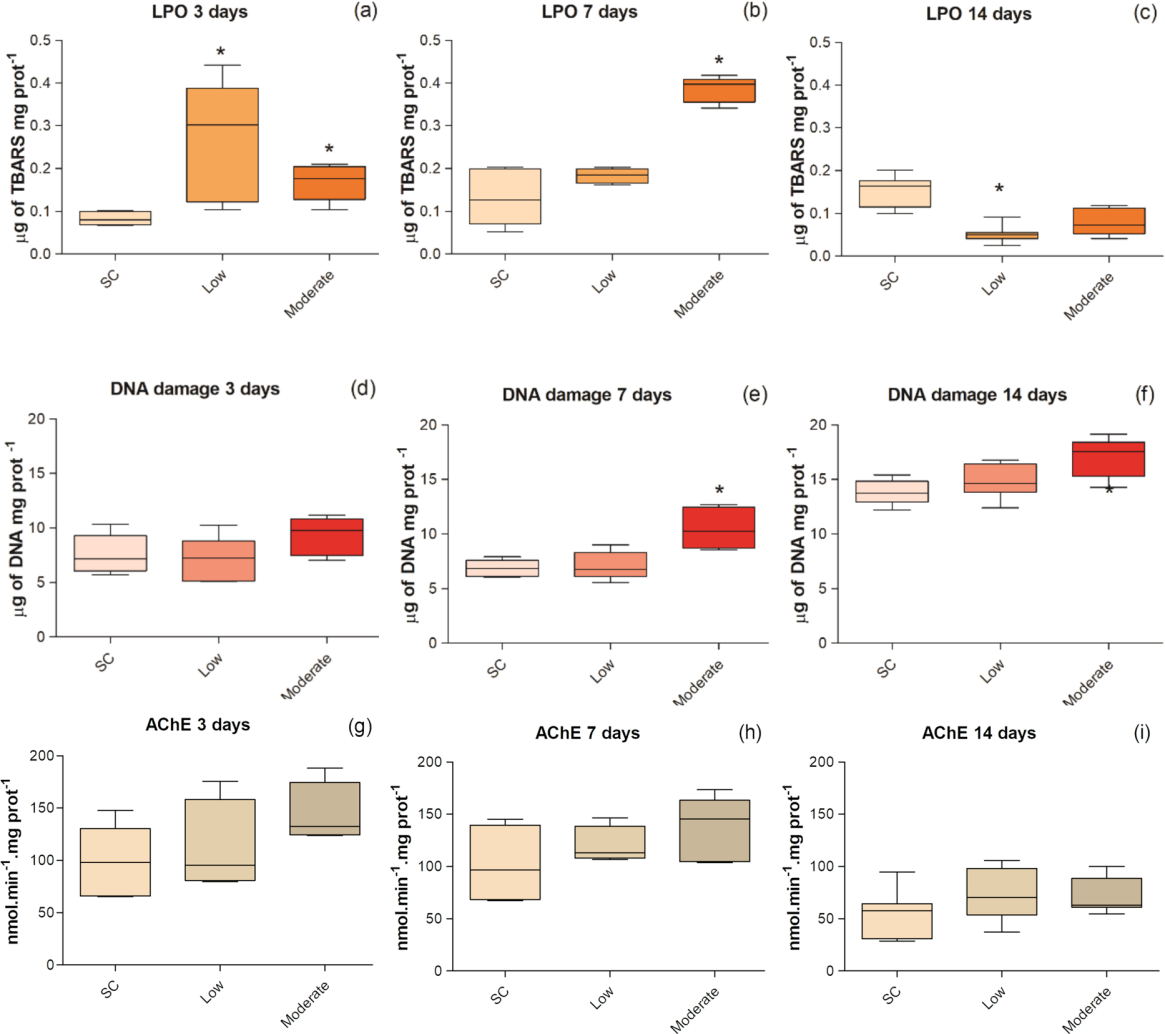
Levels of LPO (a, b and c) and DNA damage in the liver (d, e and f), and activities of AChE (g, h and i) in the axial muscle of *P. reticulate* exposed to different levels of sediment metal contamination and exposure times. Data are presented as boxplots with limits indicating 25th and 75th percentiles; a line inside the box marks the median value; markings below and above the box indicate the 10th and 90th percentiles. The asterisks mean statistical differences compared to the control sediment (*p* < 0.05). SC = sediment control, Low = low metal contamination, Moderate = moderate metal contamination

The levels of LPO, DNA damage and the activity of AChE in *P. reticulata* exposed to sediment control, low and moderate levels of metal contamination in the sediment for 3, 7, or 14 days are shown in Fig. 3.

LPO levels in *P. reticulata* liver tissue (Fig. 3), when compared to the control treatment, were higher at low and moderate contamination for the 3-day exposure (KW; *H*(2) = 10.11; *p* = 0.006); after 7 days, only the individuals exposed to moderate contamination had higher LPO levels than the control (KW; *H*(2) = 9.23; *p* = 0.01). At 14 days of exposure, in turn, LPO levels in fish exposed to low and moderate metal contamination were lower than those obtained in the sediment controls (statistically significant only for low contamination) (KW; *H*(2) = 14.46; *p* = 0.001).

The results of DNA damage analysis in *P. reticulata* liver tissue revealed a monotonic increase in relation to the level of metal contamination (Fig. 3). However, statistical differences in relation to the sediment control were observed only in individuals exposed to moderate contamination, and only in tests with longer exposure times, i.e., 7 days (ANOVA; *F*(2,12) = 10.69; *p* = 0.002) and 14 days (ANOVA; *F*(2,18) = 9.68; *p* = 0.001).

The results of AChE activity in the axial muscle of the individuals did not show significant differences between the contamination levels at any of the exposure times (3, 7 or 14 days) (KW; *H*(2) = 4.90; *p* = 0.09; *H*(2) = 1.72;  = 0.42; *H*(2) = 4.01; *p* = 0.14, respectively) (Fig. 3). However, a trend of monotonic increase from the lowest to the highest levels of contamination can be observed at any of the exposure times.

#### Liver histopathology

**Fig. 4:**
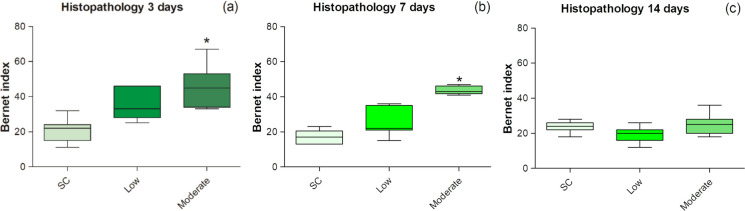
Histopathological injury index of *P. reticulata* liver, at three different time: 3 days (a), 7 days (b), and 14 days (c). The limits indicate the 25th and 75th percentiles; the line inside the box represents the median; markings below and above the box indicate the 10th and 90th percentiles. An asterisk indicates a statistical difference relative to the control (p < 0.05). SC = control sediment; Low = low metal contamination; Moderate = moderate contamination

**Fig. 5:**
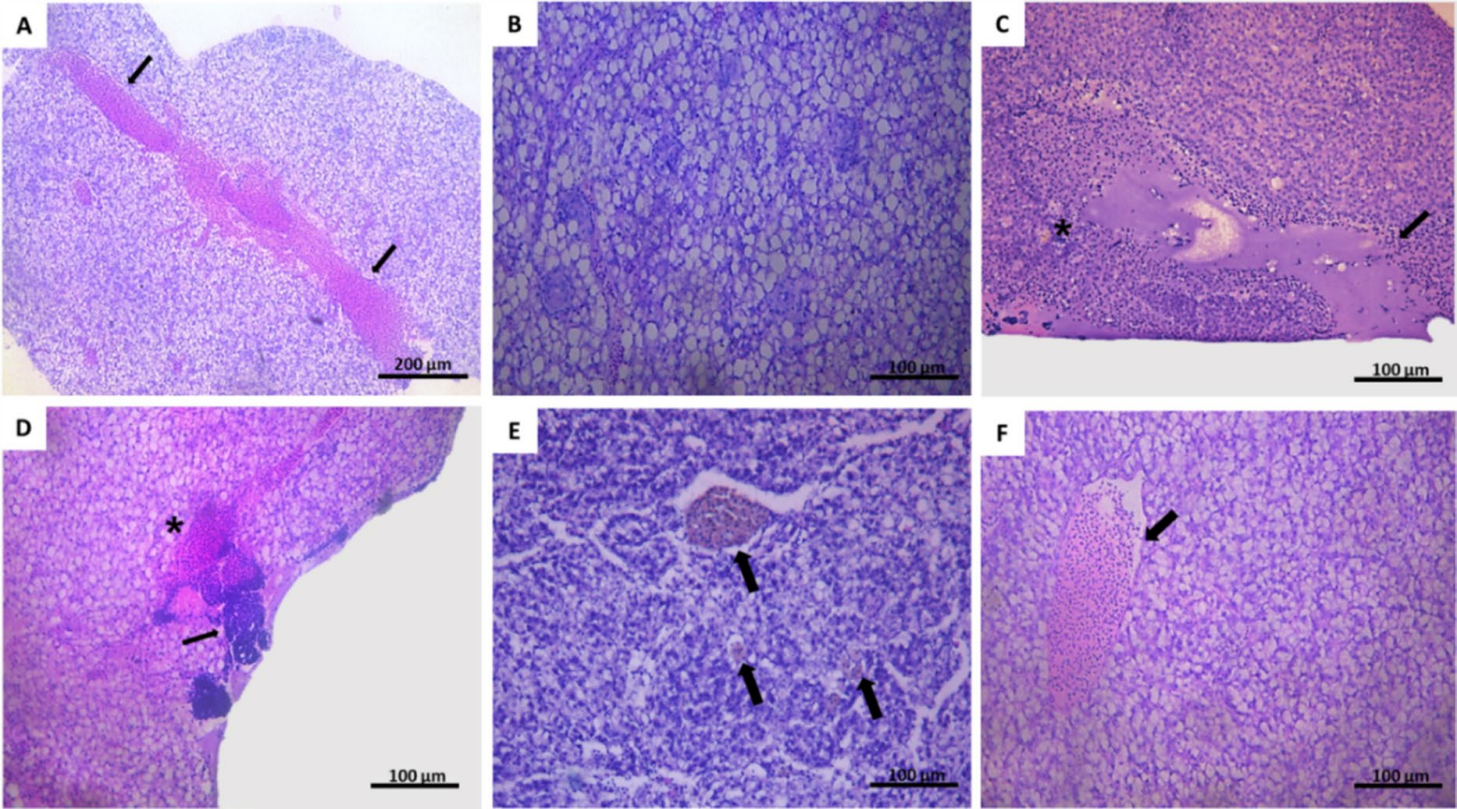
Histopathology of *P. reticulata* liver exposed to metal-contaminated sediments. (A) change in the circulatory system, showing extensive hemorrhage (H) (↑); (B) pronounced hepatic steatosis; (C) large focus of necrosis of the liver parenchyma (↑), where there is loss of cell delimitation (cell rupture), with changes in the nucleus and shape of the hepatocytes, around the lesion it is possible to see infiltration of leukocytes (*); (D) hemorrhage (*) followed by a large focus of leukocyte infiltration (↑); and (E) presence of melanomacrophage centers (↑) and (F)–vascular congestion (↑)

Hepatic histopathological damage, quantified in an integrated manner using the Bernet index, was significantly greater than the control at the moderate contamination level after 3 (ANOVA; *F*(2,18) = 12.92; *p* = 0.0003) and 7 days (KW; *H*(2) = 15.51; *p* = 0.0004) (Fig. 3). However, after 14 days of exposure, histopathological results in organisms exposed to metals showed no significant differences in relation to organisms from the sediment control (KW; *H*(2) = 5.00; *p* = 0.08) (Fig. 4). The most frequent lesions in all groups were infiltration, necrosis, apoptosis and steatosis (Fig. 5).

#### Integration of biomarker responses

**Fig. 6:**
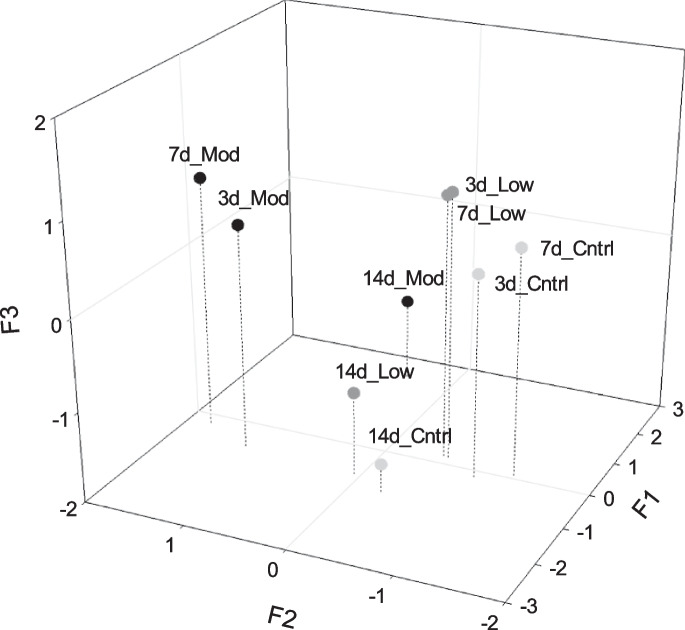
Factor scores estimated for each of the different levels of metals in sediments following exposure durations of 3, 7, and 14 days. Cntrl = sediment control, Low = low metal contamination, Mod = moderate metal contamination

Integration through FA/PCA resulted in three new variables (factors), which together corresponded to 84.76% of the variance present in the original data matrix (Table 3). Factor 1 (F1) explained 43.83% of the variation in the original data and revealed positive associations between GSH and body burdens of Pb, Cu, and Zn and negative associations with Hg body burdens. These variables together show comparatively higher values in organisms exposed to moderate contamination for 14 days (Fig. 6).

**Table 3:** Factor loadings (after Varimax rotation) of the Factor Analysis with extraction by principal components analysis applied on the original dataset (loadings >|0.5| are presented in bold text). The variances of the principal factors are given in percentage of the total variance in the original data matrices

Original variables	Factors		
	F1	F2	F3
AChE	−0.11	0.40	**0.89**
DNA	0.27	0.39	**−0.86**
GSH	**0.97**	−0.10	0.08
LPO	−0.14	0.47	**0.58**
GST	0.04	**0.55**	**0.53**
GPX	−0.16	−0.19	−**0.86**
Histopathological index (Bernet)	−0.12	**0.78**	0.46
Cu body burden	**0.91**	0.17	−0.34
Pb body burden	**0.76**	**0.61**	−0.11
Zn body burden	**0.90**	0.23	−0.24
Hg body burden	−**0.56**	−0.16	−0.16
Cu sediment	0.35	**0.90**	0.01
Pb sediment	0.38	**0.91**	0.03
Zn sediment	0.04	**0.94**	0.26
Hg sediment	0.38	**0.87**	0.11
Explained variance	44.83%	28.71%	11.22%

Factor 2 (F2) explained 28.71% of the variance in the original data and revealed a positive association between GST, histopathological index, Pb body burden and metals in sediments (Cu, Pb, Zn, and Hg). These variables are only important (higher values) at moderate concentrations of metals at the three exposure times tested (3, 7, and 14 days) (Fig. 6).

Factor 3 (F3) explained 11.22% of the variance in the original data and revealed positive associations between AChE, GST, and LPO with higher scores at 3 days (only in low metal concentration) and 7 days of exposure (both low and moderate metal contamination). Another positive association presented in F3 was GPX with DNA damage, while AChE, GST, and LPO were negatively associated with this factor. GPX and DNA damage show relatively higher values after 14 days of exposure regardless of the contamination treatment (control sediment, low, and moderate concentrations of metals) (Fig. 6).

## Discussion

In biologically based quality assessment studies and biomonitoring of aquatic environments, responses to severe pollution tend to be clear and unambiguous (Burton, 2010; Long et al., 1995). The results of this study reinforce this pattern, as evidenced by the acute impact observed in *P. reticulata* (100% mortality) within a short exposure period (6 h) at the highest metal contamination level. In contrast, responses to low or moderate pollution levels are often more susceptible to confounding factors, particularly in naturally variable environments such as estuaries. In such cases, the evaluation of sublethal responses becomes as important as it is challenging. In the present study, biological responses were also observed at moderate sediment metal concentrations (i.e., between TEL and PEL), although they were less straightforward than those recorded under extreme contamination. At low concentrations (below TEL), the responses were subtle, transient, or even absent.

*P. reticulata* tended to accumulate increasing amounts of Cu, Pb, and Zn over time, with bioaccumulation patterns depending on sediment contamination levels, exposure duration, and the specific metal accumulated. At low contaminant levels (below sediment threshold effect levels), measurable accumulation of Cu and Zn occurred in the short term, as observed after 3 days. However, after 7 and 14 days, body burdens decreased, suggesting that excess metals were actively eliminated, although this pattern was evident only at the lowest contamination level. In contrast, Hg was excreted under both low and moderate contamination starting as early as 3 days of exposure, and tissue body burdens remained below control levels after 7 and 14 days, unlike Cu and Zn, which were initially accumulated and subsequently eliminated.

Metal bioaccumulation represents the net balance between uptake and elimination processes (McGeer et al., 2004). Thus, lower Cu, Zn, and Hg body burdens in organisms exposed to low-contamination sediments may result from both active elimination and reduced uptake under these conditions. Cellular uptake mechanisms may be shared among different metals, allowing ions such as Cu^2^⁺ and Zn^2^⁺ to compete for transport into cells (Li et al., 2024). Similarly, excretion pathways—including biliary, fecal, and branchial routes, with urinary elimination generally playing a minor role—are often shared among metals (Wang, 2016). Consequently, interactions between trace metals can occur during the elimination process, potentially producing antagonistic effects, such as the inhibitory effect of Zn on Cd accumulation in *Tilapia nilotica* via enhanced Cd elimination (Kargin and Çoğun, 1999).

Although fish possess the ability to eliminate potentially harmful metals to some extent, elevated environmental concentrations often result in increased metal uptake, which may eventually exceed the organism’s detoxification capacity. In the present study, Cu and Zn exhibited progressive accumulation over time under moderate contamination. A similar pattern was observed for Pb but, in this case, even at low sediment contamination levels. These metals enter fish and other vertebrate cells primarily through specialized transporters located in the gills and intestines (Lee et al., 2019; Chandrapalan et al., 2021; Kwong, 2024). Lead displays chemical properties similar to calcium, entering cells through the same transport mechanisms and being deposited mainly in bone tissue, which has a high calcium content (WHO, 1995; Riva et al., 2006; Dai et al., 2011; Łuszczek-Trojnar et al., 2013). In addition, due to atomic properties such as a relatively large ionic radius and high electronegativity (Verstraeten et al., 2008), Pb forms more stable complexes with proteins compared to other metals (Garza et al., 2006), thereby exhibiting a greater capacity for bioaccumulation (Lee et al., 2019). For these metals, particularly for Pb, the physiological mechanisms responsible for elimination in *P. reticulata* appear insufficient to counterbalance uptake. These findings are concerning, as metal accumulation can occur even at low sediment concentrations. In addition, the results show that Pb appears to be largely insensitive to homeostatic regulation even at low concentrations, increasing consistently over time and with rising environmental levels, thereby posing a potential chronic risk and making it particularly relevant for biomonitoring purposes.

The bioaccumulation of metals, although having concerning environmental consequences such as the availability of these elements to higher trophic levels, does not necessarily cause effects on the health of the organisms that accumulate them. This occurs because trace metals can be stored in nontoxic forms (e.g., associated with intracellular insoluble granules, or heat-stable proteins including metallothioneins) (Sappal and Kamunde, 2009; Kamunde & MacPhail, 2011; Le Croizier et al., 2019), and organisms can also present response mechanisms to their harmful effects. A protective mechanism observed in the present study was the likely sequestration of excessive amounts of metals by GSH in the liver. GSH is a nonprotein cytosolic thiol with a strong affinity for most metals, and its primary function is to protect cells from toxicity (Kovářová & Svobodová, 2009). The FA/PCA analysis showed that Cu, Pb, and Zn body burdens were associated with hepatic GSH levels. This association may result from the role of GSH as a metal scavenger, but it is also possible that GSH was acting in the defense against oxyradicals (Meister & Anderson, 1983), since the generation of reactive oxygen species is a common mechanism of toxicity induced by divalent cationic metals (Balali-Mood et al., 2021).

The results of the present study revealed a strong association between metal concentrations in sediments (i.e., Cu, Zn, and Pb) and histopathological alterations in the liver of *P. reticulata* (liver histopathological index, HI). This indicates that sediment metal concentrations between the TEL and PEL thresholds, although considered to represent moderate contamination, are sufficient to elicit biological responses at the histological level. Histopathological alterations represent an intermediate level of biological response between biochemical and individual-level effects (Gusso-Choueri et al., 2022). The most common hepatic lesions observed in organisms exposed to metal-spiked sediments in the present study were inflammatory infiltration, necrosis, apoptosis, and steatosis. Similar histopathological alterations have also been reported in fish exposed to metals (Gusso-Choueri et al., 2022; Rajeshkumar et al., 2017; Costa et al., 2009). These hepatic pathologies can lead to severe and potentially irreversible liver damage.

The association of these effects with metal concentrations in sediments rather than with tissue metal levels challenges the simplistic expectation that higher environmental exposure results in greater bioaccumulation, and that higher bioaccumulation leads to stronger effects. In the present study, as previously discussed, metal body burdens were primarily associated with GSH levels (a biochemical response), whereas metal concentrations in sediments were associated with the histopathological index (a histological response). A similar pattern, where more severe histopathological effects were more strongly related to sediment metal concentrations than to bioaccumulated metals, has also been reported in estuarine fish exposed to mining waste (Gusso-Choueri et al., 2022). The stronger association of hepatic tissue lesions with sediment contamination levels than with tissue metal concentrations poses challenges to the use of tissue metal burdens as proxies of risk, at least under exposure to low to moderate trace metal concentrations. In such cases, biologically based assessments and monitoring should not rely exclusively on tissue residue levels but should instead integrate multiple lines of evidence of exposure, in addition to lines of evidence of effects, to achieve a more robust evaluation of ecotoxicological risks.

The stronger associations between the most severe effects (i.e. histological damage) and levels of contaminants in the sediment, rather than in the tissues, may be the result of two nonexclusive processes: (i) on the one hand, individuals with higher levels of metals in tissues and milder biological responses may have presented a pollutant-sequestration system of detoxification, such as the aforementioned intracellular inclusion bodies (e.g., metal-rich granules) and heat-stable proteins (e.g., metallothioneins), causing metals to bioaccumulate but not cause toxicity (refer to Vijver et al., 2004 and Adams et al., 2011 for reviews); (ii) on the other hand, organisms that presented more severe biological effects but lower levels of metals in their tissues probably began a metal excretion process after internal levels reached a certain toxic threshold (Adams et al., 2011). Consequently, a temporal mismatch may exist between the comparatively faster dynamics of metal regulation and the longer-lasting manifestation of histopathological effects. Even so, since Pb body burden was also associated with histopathological lesions, it is likely that this trace metal made the greatest contribution to the observed liver damage. As discussed earlier, Pb accumulated over time in both low and moderate-contamination treatments, suggesting that this element is not as efficiently detoxified as the other metals investigated in this study.

Results also revealed associations between the biochemical biomarkers AChE, GST, and LPO on one hand, and GPx and DNA damage on the other. None of these groups was associated with metal concentrations in sediments or in bioaccumulated forms. The increase in hepatic LPO levels in metal-exposed organisms after 3 days of exposure indicates that the antioxidant system of *P. reticulata* was insufficient to neutralize reactive oxygen species (ROS) during short-term exposure. At the same early exposure time, the observed reduction in GSH levels due to metal exposure may explain the lower activities of GPx and GST, as both enzymes depend on GSH for their catalytic function (Oliva et al., 2014; Pereira et al., 2010). This GSH depletion suggests that the liver was unable to promptly eliminate oxyradicals. Similar GPx inhibition has been reported in fish exposed to metals (Liu et al., 2006; Banni et al., 2011; Qu et al., 2014).

Although GSH levels increased over the subsequent exposure periods, particularly under moderate contamination, GPx activity remained inhibited, suggesting that its antioxidant capacity may have been overwhelmed by the accumulation of ROS, thereby reflecting a potential failure of the antioxidant defense system. This interpretation is supported by the observed patterns of LPO at early and mid-term exposure, and by DNA damage at intermediate and longer exposure durations. In contrast to GPx, GST and GSH exhibited a more effective antioxidant response, with activity increasing after 7 days and either maintaining active defenses (GSH) or returning to control levels (GST) by day 14. Although these antioxidant biomarkers were not grouped in the FA/PCA due to their differing mid- and long-term dynamics, univariate analyses suggest that they operate in a biochemically coordinated manner.

Recovery of antioxidant activity after 7 days likely contributed to the marked reduction in hepatic LPO levels and the restoration of liver histoarchitecture in *P. reticulata* after 14 days of exposure, reversing lesions observed at earlier time points. ROS are known to induce lipid peroxidation (van der Oost, 2003), which can diffuse intracellularly (Rajeshkumar et al., 2017) and cause tissue-level damage (Fatima et al., 2015; Javed et al., 2016, 2017). Recovery was also observed for AChE activity in axial muscles, which initially tended to increase under low and moderate metal concentrations, likely due to interactions of metals with acetylcholine receptors, acetylcholine accumulation, and subsequent compensatory AChE induction (De Lima et al., 2013; Bainy et al., 2006; Romani et al., 2003; Almeida et al., 2024). Together, these results reflect physiological acclimation to non-lethal metal exposure, consistent with the long-established damage-repair model (McDonald & Wood, 1993; McGeer et al., 2007). Such acclimation involves modifications in metal uptake and distribution, increased synthesis of metal-binding molecules (e.g., metallothioneins and GSH), as observed in the present study in organisms exposed to moderate contamination levels, and enhanced elimination (Grosell et al., 1997; McGeer et al., 2007; Adeyemi & Klerks, 2013), observed in organisms exposed at lower contamination levels. This indicates coordinated recovery mechanisms across biochemical, neurophysiological, and tissue levels.

Although several biomarkers indicated recovery over time, DNA damage in the liver of *P. reticulata* was the only parameter that continued to increase with longer exposure. This may reflect initial inhibition of the antioxidant system, evidenced by decreased GSH levels and reduced GPx and GST activities, particularly under moderate contamination, as excessive ROS can induce DNA strand breaks (Livingstone, 2003). However, the persistence of DNA damage despite recovery of antioxidant defenses suggests that other mechanisms, such as the formation of DNA adducts through intercalation or covalent binding, may also contribute (Javet et al., 2017, Bhat et al., 2025). Regardless of the mechanism, the occurrence of DNA damage at moderate sediment metal concentrations is concerning, as it may lead to cell death, mutations, genotoxicity, and long-term effects on reproduction and development, including embryonic aberrations, reduced hatching rates, impaired gamete development, and diminished physical capacity (Sahlmann et al., 2017). Considering that *P. reticulata* is a species tolerant of stressful environments, the occurrence of toxic responses even under moderate contamination conditions raises concerns about the implications of these findings for more sensitive species, highlighting the need for similar studies with such taxa to better understand the extent of ecological risks associated with trace metal-contaminated sediments, even at concentrations considered relatively low.

From the perspective of using biomarkers in *P. reticulata* for biologically based quality assessment and biomonitoring of estuarine environments, some responses to metal contamination showed sensitivity and stability over time. Non-protein thiol levels (GSH) in the liver, for example, were affected by sediment metal contamination from the beginning of exposure, either showing inhibition at a shorter exposure period or an increase after a longer exposure. GSH also showed a strong association with metal body burdens, indicating a consistent response to internal metal levels in the fish. DNA damage in the liver similarly demonstrated temporal consistency and sensitivity to moderate contamination levels tested in this study. This demonstrates that even a species tolerant of degraded environments can exhibit sensitivity to contamination when assessed using a set of sensitive biomarkers guided by the toxicodynamic mechanisms of the stressors.

In contrast, TBARS (LPO) levels and hepatic GST activity showed pronounced responses during short-term and medium-term exposures but failed to maintain sensitivity over time. Muscle AChE activity, which was associated with both LPO and GST in the multivariate analysis, followed a similar overall trend, although univariate tests did not reveal statistically significant differences. These findings indicate that, while such biomarkers can provide valuable information when integrated into a multibiomarker approach to assess organism health, their responses exhibit limited temporal linearity under low-to-moderate contamination, emphasizing the need to account for exposure duration in data interpretation.

Finally, the histopathological index also demonstrated good sensitivity during early and mid-term exposure, but its responsiveness declined after 14 days. Its strong association in the FA/PCA with sediment contamination and accumulated Pb suggests that it represents a promising sublethal biomarker for biologically based quality assessment and biomonitoring of mildly contaminated estuarine environments. However, caution is warranted in the interpretation of longer-term exposures, given the non-linear temporal responses and potential recovery toward homeostasis, particularly in cases such as the present study, where some histopathology indicate partial recovery while others continue to reflect progressive stress with increasing exposure time and contamination level.

## Conclusion

The results of the present study highlight the importance of considering both exposure duration and contamination levels when interpreting biomarker responses in ecotoxicological studies, particularly in environments with low to moderate contamination. The coexistence of early responses, partial recovery, and persistent effects (such as DNA damage) suggests the presence of physiological compensation strategies with latent biological costs.

The differential sensitivity and recovery patterns observed in *P. reticulata* across multiple biomarker levels emphasize the limitations of assessments based solely on short-term exposures or single biomarkers, and stress the need to account for temporal dynamics in biomarker-based monitoring. Furthermore, the occurrence of biological effects even at low to moderate contamination levels supports the need for more precautionary thresholds in sediment-quality guidelines. Integrating multilevel biomarkers into environmental monitoring programs can enhance the early detection of sublethal effects and provide a more comprehensive understanding of ecosystem health. Thus, the use of *P. reticulata* as a biomonitor, combined with a multibiomarker approach, represents a valuable tool for improving the assessment, monitoring, and management of environmental quality in aquatic ecosystems.

## Supplementary Information

Below is the link to the electronic supplementary material.

ESM 1 (DOCX 863 KB)

## Data Availability

Data will be available as requested.
