# Supplementary material for: Time and contamination level dependence of metal bioaccumulation and multibiomarker responses in fish: implications for biomonitoring
Source: Environ Monit Assess. 2026 Mar 23;198(4):342. doi: 10.1007/s10661-026-15181-9 (PMC13009005; doi:10.1007/s10661-026-15181-9)
Supplement: Supplementary file 1 — (DOCX 863 KB) [file 10661_2026_15181_MOESM1_ESM.docx]

**Table S1.** Results (mean and standard deviation) of biomarkers evaluated in the liver or muscle of *P. reticulata* exposed in T0 and control treatments. GST, GPX and AChE values are expressed in nmol min^-1^ mg prot^-1^, GSH in nmol mg prot^-1^, LPO in µg of TBARs mg prot^-1^, DNA damage in µg of DNA mg prot^-1^.

| **Treatament** |  |  | **Liver** | | | | | | | | | | |  | **Axial muscle** |
| --- | --- | --- | --- | --- | --- | --- | --- | --- | --- | --- | --- | --- | --- | --- | --- |
|  |  |  | **GSH** |  | **GST** |  | **GPX** |  | **LPO** |  | **DNA** |  | **Bernet index** |  | **AChE** |
| **T0** |  |  | 4.68±1.93 |  | 11.12±2.67 |  | 6.40±1.03 |  | 0.22±0.01 |  | 8.17±2.15 |  | 27.75±5.50 |  | 87.59±36.55 |
| **Sediment control** | **3d** |  | 4.08±0.48 |  | 8.56±2.01 |  | 6.17±0.65 |  | 0.08±0.02 |  | 7.67±1.74 |  | 21.29±6.78 |  | 98.14±34.51 |
| **Sediment control** | **7d** |  | 3.76±0.44 |  | 6.95±3.56 |  | 5.16±0.9 |  | 0.13±0.07 |  | 6.86±0.77 |  | 17±3.71 |  | 101.53±38.49 |
| **Sediment control** | **14d** |  | 0.67±0.23 |  | 5.75±1.36 |  | 9.95±2.92 |  | 0.15±0.04 |  | 13.85±1.12 |  | 23.71±3.35 |  | 52.38±23.9 |
